# Application of holographic display in radiotherapy treatment planning II: a multi‐institutional study

**DOI:** 10.1120/jacmp.v10i3.2902

**Published:** 2009-05-28

**Authors:** James C H Chu, Xing Gong, Yang Cai, Michael C Kirk, Thomas W Zusag, Susan Shott, Mark J Rivard, Christopher S Melhus, Gene A Cardarelli, Amanda Hurley, Jaroslaw T Hepel, Josh Napoli, Sandy Stutsman, Ross A Abrams

**Affiliations:** ^1^ Rush University Medical Center Chicago IL; ^2^ Tufts‐New‐England Medical Center Boston MA; ^3^ Rhode Island Hospital Brown Medial School Providence RI; ^4^ Actuality Medical, Inc. Bedford MA

**Keywords:** treatment planning, radiation therapy, 3D display, volume imaging, virtual reality

## Abstract

We hypothesized that use of a true 3D display providing easy visualization of patient anatomy and dose distribution would lead to the production of better quality radiation therapy treatment plans. We report on a randomized prospective multi‐institutional study to evaluate a novel 3D display for treatment planning.

The Perspecta® Spatial 3D System produces 360° holograms by projecting cross‐sectional images on a diffuser screen rotating at 900 rpm. Specially‐developed software allows bi‐directional transfer of image and dose data between Perspecta and the Pinnacle planning system.

Thirty‐three patients previously treated at three institutions were included in this IRB‐approved study. Patient data were de‐identified, randomized, and assigned to different planners. A physician at each institution reviewed the cases and established planning objectives. Two treatment plans were then produced for each patient, one based on the Pinnacle system alone and another in conjunction with Perspecta. Plan quality was then evaluated by the same physicians who established the planning objectives. All plans were viewable on both Perspecta and Pinnacle for review. Reviewing physicians were blinded to the planning device used. Data from a 13‐patient pilot study were also included in the analysis.

Perspecta plans were considered better in 28 patients (61%), Pinnacle in 14 patients (30%), and both were equivalent in 4 patients. The use of non‐coplanar beams was more common with Perspecta plans (82% vs. 27%). The mean target dose differed by less than 2% between rival plans. Perspecta plans were somewhat more likely to have the hot spot located inside the target (43% vs. 33%). Conversely, 30% of the Pinnacle plans had the hot spot outside the target compared with 18% for Perspecta plans. About 57% of normal organs received less dose from Perspecta plans. No statistically significant association was found between plan preference and planning institution or planner.

The study found that use of the holographic display leads to radiotherapy plans preferred in a majority of cases over those developed with 2D displays. These data indicate that continued development of this technology for clinical implementation is warranted.

PACS numbers: 87.55.D

## I. INTRODUCTION

Ionizing radiation is an effective treatment modality for cancer. A sufficient dose of radiation must be delivered to the target volume to achieve the therapeutic objective while respecting normal organ dose limits. Finding the optimal method to deliver the desired treatment in a given patient has been a well‐recognized problem since the practice of radiation therapy began nearly a century ago, when “erythema dose” was the metric used to quantify the radiation delivered. Much of the focus of radiation therapy development over the years has been on devising methods and technologies to improve the therapeutic ratio. A wide variety of treatment units are now available to provide safely and consistently various types of radiations (photons, electrons, or heavier particles) with sophisticated geometric positioning, collimation, and even the ability to vary the radiation fluence within the aperture of a particular beam.^(1)^ To fully utilize the capabilities of modern radiotherapy equipment to direct beams and shape high‐dose volumes, patient imaging that shows the spatial relationship of tumors and critical normal structures must be available during the treatment planning process.

Radiotherapy treatment planning, a process during which radiation beams are selected to avoid as much critical tissue as possible while delivering high doses to the tumor, is one of the critical steps of a successful radiation treatment program. The planning process makes heavy use of volumetric imaging of patient anatomy from CT, MRI, PET, SPECT or other modalities to determine beam orientations, shapes, and intensity for the patient's radiation treatment. Radiation dose distributions are calculated within the patient's anatomy and compared during evaluation of rival treatment plans. Sophisticated computer algorithms and technologies are used to perform calculations and display the results, providing users with an understanding of dose delivery in three dimensions. Even with these technologies, selection of beam arrangements to generate the optimal plan is not trivial. Among the complexities of beam selection and plan evaluation, one of the most vexing is that most current display technologies can show only two‐dimensional images to represent the beam orientation and dose coverage that exist in the three‐dimensional world. Although 2D representation of the 3D world is viewed by each eye, a variety of depth cues are available to afford true 3D visualizations.^(2)^ These depth cues are not readily available in the display on a computer screen. Multi‐planar 2D and pseudo 3D displays on a flat computer screen provide only limited depth cues and, therefore, may not readily present the three dimensionality of an object. A true 3D stereoscopic display, which allows almost all depth cues, may provide the 3D perspective of an object more quickly and effectively.^(^ ^3^ ^,^ ^4^ ^)^

The Perspecta® Spatial 3D System, a new volumetric display device recently developed by Actuality Medical Inc. (Bedford, MA, USA), is a system that generates true, volume filling, 3D imagery that can be inspected from any viewpoint rather than upon a planar surface.^(5)^ More importantly, the Perspecta images are autostereoscopic – viewable from almost any angle with the unaided eye (without any goggles). This can be particularly useful for radiotherapy treatment planning, as physicians and planners can view the image together while discussing various planning strategies. We hypothesized that, except when standard beam arrangements are used, such a true 3D display providing easy visualization of patient anatomy and dose distribution might lead to the production of better quality radiation therapy treatment plans. To test this hypothesis, we have conducted a randomized prospective multi‐institutional study using the new Perspecta 3D display. This paper reports the results from this study.

## II. MATERIALS AND METHODS

### A. Display System

The Perspecta system v1.9 produces 25 cm (10 inch) diameter, 360°‐viewable 3D hologram‐like imagery by projecting multiple cross‐sectional 2D patterns (slices) onto a diffuser screen rotating at about 900 rpm. One hundred and ninety eight (198) cross‐sectional slices of 768×768pixels resolution are displayed over 180° during rotation. Since the volume is refreshed at 30 Hz, this equates to approximately 6,000 slices/sec for a total of 3.5 Gpixels/sec. The slices optically originate in three digital mirror devices based on the Digital Light Processing™ technology from Texas Instruments Inc. (Plano, TX, USA). The slices are projected to the rotating screen by a custom optical relay system.

The 3D images appear to be floating in space within a 61 cm (24 inch) dome due to visual persistence. Proprietary optics and computer software are used to stabilize and maintain focus of the image. The 50/50 reflectance and transmission property of the diffuser screen, in conjunction with an omnidirectional diffusion profile, makes the images appear to be semi‐transparent. This feature is particularly useful for this study because structures enclosed by a particular isodose surface are still visible during treatment plan evaluation. More detailed descriptions of the Perspecta system were presented previously.^(^ ^6^ ^,^ ^7^ ^)^

**Figure 1:**
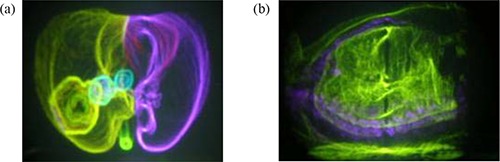
Perspecta autostereoscopic display of images transferred from Pinnacle planning system. Fig. 1(a) shows a region of interest (ROI) based image. Individual ROIs can be tagged with different colors. Right and left lungs are in green and purple respectively. Tumor is bright green in right lung. Nodal disease is in blue. Fig. 1(b) represents CT‐based image. Different colors can be assigned to specific ranges of CT densities. Here purple is assigned to bone.

**Figure 2:**
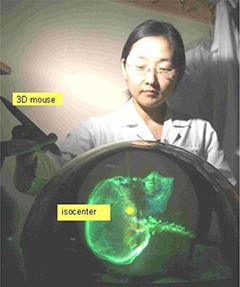
Hologram image‐based beam placement. Radiation beams are placed by the use of a 3D mouse. The handheld device moves the isocenter within the imagery.

Hardware and software interfaces were developed to allow bidirectional data transfer between the Perspecta display system and Pinnacle^3^ treatment planning system (Philips Medical Systems Inc. Madison, WI, USA). Planning data from Pinnacle^3^, such as CT images, regions of interest (ROI), reference points (RP), beam geometries, and dose distributions can be easily transferred and displayed on Perspecta by activating a single synchronization control. Figure 1 shows the Perspecta display of ROI (Fig. 1a) and CT (Fig. 1b) based images for a lung cancer patient transferred from Pinnacle. Different ROIs can be distinguished by assigning different colors to them. Different colors can also be assigned to various ranges of density windows in the CT based display. For example, in Fig. 1b, purple is used to display the bone window. The display system also includes a 3D mouse (PHANTOM® Omni™, SensAble Technologies Inc., Woburn, MA, USA) which moves a cursor within the imagery via an articulated arm. By clicking a button on the arm, any point can be defined within the display. Figure 2 shows an isocenter as it is being placed on the Perspecta display using the 3D mouse. With the isocenter defined, the 3D mouse can also be used to specify beam orientations by clicking on any point along the beam axis. Isocenter, RPs, and beam geometries (including gantry and couch angles) defined by the 3D mouse on Perspecta can be transferred to Pinnacle via the same synchronization device. Custom software calculates gantry and couch angles automatically for any non‐coplanar beam defined on Perspecta. The same software also checks the beam setup parameters against a collision avoidance table and only allows selection of deliverable beams. In addition, Perspecta has implemented planning tools, such as a 3D ruler, auto‐surround for field shaping, point dose display, and real time display of selected ROI volumes intersected by particular beams, to facilitate the hologram based treatment planning. It is fairly easy to examine the adequacy of target coverage and the volume of critical organ tissue irradiated since the entire beam border is visible in this display.

### B. Study Design

Thirty‐three patients treated at three institutions with 3D conformal or intensity modulated radiation therapy (IMRT) treatment were included in the study. The data include 12 brain, 10 lung, and 11 abdomen/pelvis cases. CT images, target volumes, and critical normal structures generated at the originating institution were used for this study. The patient CT and structure data were rendered anonymous and the study was approved by the institutional review boards (IRB) at all three institutions. Patients were randomly assigned to planners who were not at the patient's originating institution. This process ensured that the planning institution and originating institution were not the same for each patient. To reduce possible planning bias, the process also assured that Perspecta and Pinnacle plans for each patient were produced by two different planners. A physician at each institution reviewed the assigned cases and established planning objectives. The objectives were specific to each case, including target coverage and normal organ sparing requirements and priorities among them. Two treatment plans were then produced for each patient, one produced solely on the Pinnacle system and another produced in conjunction with Perspecta. Planners were free to use all available tools, including both 2D and pseudo 3D rendering tools. There was no restriction regarding the number of beams used for either Pinnacle or Perspecta plans. Plans were then evaluated by the same physicians who established the planning objectives for the specific case. This was necessary to ensure that the same guidelines were used for treatment planning and plan evaluation. All plans were viewable for evaluation on both Perspecta and Pinnacle. While the process of choosing the “better” plan was often subjective, the preferred plan may have had better target coverage or normal tissue sparing reflecting the priority and objectives defined for the case. The dose volume histogram (DVH) and the equivalent uniform dose (EUD) for target volumes and various ROIs were also calculated as metrics for plan analysis. The EUD, normalized to the prescription (nEUD), was used to analyze dose delivered to the target volume. In addition, we also recorded the length of time required for planning, the use of non‐coplanar beams, and the location of high dose regions. Physicians were free to use whatever evaluation tools were available and were blinded to the planning device used during their review.

Data from this study were combined with that from a non‐randomized pilot study^(8)^ which included 12 previously treated brain cancer patents replanned at two institutions and one lung patient replanned at a third institution. The reviewers could rate a particular plan better than or equivalent to the rival plan during the pilot study; however, the option for assigning an equivalent score to both Pinnacle and Perspecta plans was not provided during the randomized study. The total number of patients in each disease site is shown in Table 1.

**Table 1:** Number of Cases Rated Better for Various Disease Sites

*Disease Site*	*Pinnacle* ^a^ *better*	*Perspecta* ^b^ *better*	*Equivalent*	*ALL*
Brain	8 (33%)	12 (50%)	4 (17%)	24
Lung	2 (18%)	9 (82%)		11
All Sites	14 (30%)	28 (61%)	4 (9%)	46

^a^ a modern treatment planning system by Philips Medical Systems, Inc.

^b^ a holographic display system by Actuality Systems, Inc.

SPSS for Windows (Version 14.0, September, 2005, SPSS Inc., 233 S. Wacker Drive, Chicago, Illinois 60606) was used for data management and statistical analysis. Because the data had distributions that were not statistically normal, nonparametric statistical methods were used for analyses. The chi‐square test of association and Fisher's exact test (when expected frequencies were too small to permit use of the chi‐square test) were used to compare independent groups with respect to percentages. The McNemar test was done to compare paired groups with respect to percentages. The chi‐square goodness‐of‐fit test was carried out to test the hypothesis of equally likely categories. The Kruskal‐Wallis and Mann‐Whitney tests were used to compare independent groups with respect to non‐categorical data, and the Friedman test was done to compare paired groups with respect to non‐categorical data. A 0.05 significance level was used for all statistical tests. No one‐sided statistical tests were done.

## III. RESULTS

The bidirectional data transfer between Perspecta and Pinnacle was successful; all beams defined by Perspecta could be calculated with Pinnacle, and all isodose plans and ROIs from Pinnacle were successfully displayed on Perspecta. No statistically significant association was found between plan preference and planning institution (p=0.43) or planner (p=0.44).

For the 46 patients in the study, Perspecta plans were rated better in 28 patients (61%) while Pinnacle plans were rated better in 14 patients (30%). The plans were considered equivalent in 4 of the brain cases included in the pilot study. The breakdown of better plans by disease site is presented in Table 1. This made Perspecta at least as good as, or better than, Pinnacle in 32 patients (70%,p=0.008). The chance of producing a better plan when using Perspecta ranged from 50% for brain patients to 82% for lung patients. When averaged over all sites, one may be twice as likely to achieve a better plan when using Perspecta in conjunction with Pinnacle rather than using Pinnacle alone.

Table 2 shows the use of non‐coplanar beams, number of planning iterations, and the length of time needed for planning. The use of non‐coplanar beams was more frequent with Perspecta (p<0.001). The Perspecta plans required more beams and time, whereas the number of iterations did not significantly differ between the two planning techniques.

**Table 2:** Planning Parameters Used for Different Plans

	*Pinnacle*	*Perspecta*	*P value*
No. of non‐coplanar plan	9/33 (27%)	27/33 (82%)	<0.001
No. of iterations			0.49
Mean	5.2	6.6	
Median	3.0	2.0	
No. of beams			0.025
Mean	4.1	4.9	
Median	4.0	5.0	
Length of planning time (minute)			0.014
Mean	37	56	
Median	29	40	

The nEUD for clinical target volumes (CTV) and the location of the hot spot relative to the target volume are presented in Table 3. The mean nEUD for Perspecta and Pinnacle plans differed by less than 2% and the median was nearly the same, indicating similar target dose delivered by rival plans. The Table also shows that Perspecta plans were more likely to have the hot spot located inside the target (43% vs. 33%), although the difference was not statistically significant (p=0.61). Conversely, 30% of the Pinnacle plans had the hot spot outside the target compared with 18% for Perspecta plans (p=0.34).

**Table 3:** Target Dose Distribution Properties

	*Pinnacle*	*Perspecta*	*P value*
nEUD^a^ for CTV^b^			0.16
Mean	0.983	1.000
Median	1.024	1.020
Hotspot location
Inside target volume	11/33 (33%)	14/33 (43%)	0.61
Edge of target volume	12/33 (37%)	13/33 (39%)
Outside of target volume	10/33 (30%)	6/33 (18%)	0.34

^a^ Normalized equivalent uniform dose, equivalent uniform dose normalized to the prescription dose.

^b^ Clinical target volume.

A total of 161 normal structures were contoured for patients in the study. Of those structures, 92 showed lower doses with the Perspecta plan (57%). The difference, however, was not statistically significant (p=0.07). Table 4 summarizes the EUD for some of those structures. Although the EUD for various normal structures was generally lower for Perspecta plans, it was statistically significant only for liver (p=0.14) and left optic nerve (p=0.002). The mean difference in $V_{25\text{Gy}}$ for liver, however, was not significant (p=0.18) between rival plans.

**Table 4:** Equivalent Uniform Dose Delivered to Normal Tissues from Perspecta and Pinnacle Plans

	*Pinnacle*	*Perspecta*	*P value*
Brain stem			0.40
Mean	32.9	33.0	
Median	33.6	34.2	
Lt. optic nerve			0.002
Mean	16.9	14.5	
Median	8.8	4.3	
Rt. optic nerve			0.20
Mean	18.7	20.4	
Median	15.8	15.3	
Spinal cord			0.64
Mean	23.0	20.6	
Median	21.5	18.3	
Optic chiasm			1
Mean	28.3	29.2	
Median	21.8	27.2	
Rt. lung			0.74
Mean	10.3	11.1	
Median	12.9	12.6	
Lt. lung			0.32
Mean	8.2	7.9	
Median	4.1	6.0	
Both lungs			0.74
Mean	10.9	12.0	
Median	9.5	10.1	
Both lungs V			0.78
Mean	15.1	16.5	
Median	12.1	15.7	
Heart			0.76
Mean	7.9	10.9	
Median	1.3	3.9	
Liver			0.014
Mean	21.1	16.5	
Median	20.7	18.8	
Liver V			0.18
Mean	19.2	11.4	
Median	19.2	12.6	
Lt. kidney			0.41
Mean	21.5	21.5	
Median	21.5	15.5	
Rt. kidney			0.41
Mean	19.6	15.3	
Median	17.7	15.2	1
Bowel
Mean	27.4	28.0	
Median	24.9	29.0	
Bladder			0.56
Mean	34.7	33.5	
Median	43.0	44.4	
Rectum			0.56
Mean	42.1	42.3	
Median	44.3	44.9	
Femoral head			0.16
Mean	25.3	18.0	
Median	25.3	18.0	
Rt. parotid mean dose			0.32
Mean	8.0	6.8	
Median	6.7	6.0	
Lt. parotid mean dose			1
Mean	4.0	3.0	
Median	4.0	3.0	

Most of the above data are equivalent dose (EUD) in Gy. Lung $V_{20\text{Gy}}$ and liver $V_{25}\;\text{Gy}$ are percent organ volume receiving the stated dose. Parotid mean dose has units Gy.

## IV. DISCUSSION AND CONCLUSION

The value and potential use of a “true 3D” display in radiation therapy was recognized more than 10 years ago when Hubbold et al.^(9)^ developed an autostereoscopic display system for radiotherapy treatment planning. The system did not achieve wide clinical use due, in part, to relatively low frame rate and limitations in displaying nested ROIs and dose volumes. With advances in computing and display technologies, there is renewed interest in true 3D display and virtual reality (VR) applications in radiation therapy. Schlaefer et al.^(10)^ designed an autostereoscopic system to facilitate beam placement for robotic radiosurgery. Patel et al.^(11)^ reported the use of a networked VR system for daily radiation therapy conferences. Beavis et al.^(12)^ and Boejen et al.^(13)^ used VR for training of radiotherapists. Shang et al.^(14)^ and Williams et al.^(15)^ reported use of VR in treatment planning for prostate cancer eliminating risk of collision and facilitating placement of non‐coplanar beams. We reported in a preliminary study that a physician's estimate of volume coverage was more accurate using the Perspecta system for a majority of ROIs during treatment planning.^(^ ^16^ ^,^ ^17^ ^)^ Rivard et al.^(18)^ also reported that the same Perspecta system may have significant potential in 4D treatment planning. Although it may be intuitive that a true 3D display may improve treatment planning efficiency and lead to better quality plans, there has been no research to demonstrate its value and identify areas it may impact significantly. To the best of our knowledge, the current study is the first randomized prospective study to compare radiotherapy plans based on a state‐of‐the‐art planning system with those based on a true 3D display.

While a comparison between plans produced by using only 3D rendering tools from Pinnacle and Perspecta may be desirable, we allowed both 2D and 3D planning tools in this study, as the 3D Pinnacle tools are not always easy to use. Another limitation of the study is that the planners may be more prone to use beam arrangements that they are familiar with when using Pinnacle; whereas with Perspecta, they may be more creative with the novel display. There was a trend in our data that Perspecta plans were better than Pinnacle plans in general, mainly due to the selection of non‐coplanar beams. For example, a Pinnacle plan used 4 coplanar oblique fields to treat an upper chest lesion; the corresponding Perspecta plan used 5 non‐coplanar beams with different combinations of couch and gantry orientations, achieving better tumor coverage and lower lung dose. In another example (a brain tumor case), the Pinnacle plan used 3 gantry angles at a fixed couch orientation; the corresponding Perspecta plan used 3 beams at 2 different couch positions, achieving similar target coverage with lower dose to optical chiasm. This type of improvement, however, did not reach statistical significance, which may be due to the small sample size and may also be a result of the subjectivity of the observers.

Perspecta plans require more time to complete than Pinnacle plans. This is contrary to the expectation that a better appreciation of the 3D spatial relationship of various ROIs would improve the planning efficiency and require less time to produce. This could be explained by lack of experience and may become less significant with more experience. While all planners were adept with the Pinnacle system, they were not nearly as familiar with Perspecta's tools and functionalities. In addition, the prototype synchronization interface was not very robust and required rather frequent computer system reboots during planning. We expect the Perspecta planning time to decrease as development of tools matures. The mean number of iterations required for producing Perspecta plans was larger than that for Pinnacle, whereas the median number of iterations was higher for Pinnacle. This was caused by the wide variation of the data. The difference in the number of iterations was not statistically significant (p=0.49).

The use of non‐coplanar beams was much more common with Perspecta. This may be one of the most significant impacts that “true 3D” display will make in radiation therapy treatment planning. Selection of radiation beam directions that maximize the separation between the tumor and critical organs is one of the most effective techniques to reduce the dose to critical normal tissues. Although the selection of beam orientation may not be as critical with intensity modulated radiation therapy (IMRT) beams, optimally oriented beams usually require fewer modulation levels.^(1)^ Coplanar beams do not always provide the optimal beam angles but they are used more often as they are easily visualized in the transverse plane and present less risk for patient/machine collision As a result, they are easier to plan. The traditionally flat screen display has a “planar bias” requiring extra effort or external models to visualize non‐coplanar beam directions; with Perspecta or VR displays 3D visualization of beam and ROI spatial relationships is quick and obvious. These devices also allow observation of possible patient/machine collision when the linear accelerator and patient are modeled and displayed.^(19)^ Planners were three times as likely to use non‐coplanar plans when using Perspecta in this study. This demonstrated that planners were comfortable using non‐coplanar beams when effective true 3D displays were available. The Perspecta display has no default viewing angle, axis, or plane, and external aids are not needed to visualize non‐coplanar beam arrangements. This may explain the increased choice of non‐coplanar beam arrangements in spite of the extra time and effort that may be required to execute such plans. While this difference was greatest in the thorax, it was present even for brain cases, which already have a high likelihood of being treated in a non‐coplanar fashion when planned with 2D visualization displays. Even here, one physician reviewing two separate non‐coplanar brain plans on the Perspecta noted that in each a beam exited through the eye, a fact that he hadn't immediately realized when reviewing the plan on a 2D display. We speculate that the cause of this adverse beam placement was the disconnect between specifying and adjusting beam geometries, and reviewing beam's eye views – two separate steps in Pinnacle. Coupled with the habit of dosimetrists of turning off low dose lines, such a placement might not get detected until the plan is finished. In contrast, defining beam geometry with Perspecta is done while viewing beam angles and divergence live in 3D, making it nearly impossible to miss that kind of error.

The use of non‐coplanar beams, however, may require additional time to set up and verify patient positions at different couch angles. The issue of treatment efficiency and patient throughput may have to be addressed if a large fraction of patients are to be treated with non‐coplanar plans.

The planners and reviewers noted that they could easily examine the relative locations of target, ROIs, and dose distributions in a 3D space by moving around the Perspecta display. This greatly facilitates the beam placement, and planners could more easily find beam angles that produce conformal plans. This may also explain why the hot spots in Perspecta plans were more likely to occur within the target volume compared with those in Pinnacle plans, as the locations of hot or cold spots along with the entire isodose surfaces and ROIs are simultaneously visible in a 3D space. The target volume coverage for Perspecta and Pinnacle plans were comparable for the majority of the cases.

Both Perspecta and Pinnacle plans delivered a wide range of doses to various normal tissues. Although Perspecta plans may be associated with slightly lower critical normal tissue dose on average, the benefit of using Perspecta in this regard is likely dependent on the particular patient geometry. Perspecta should be useful for those patients with complex geometry and requiring unconventional beam orientations.

Our current study compared radiation treatment plans produced by various planners from different institutions. One potential problem with this type of study is the possible bias from differences in individual planner skills and/or institutional expertise. The fact that our results were not associated with these factors confirmed the adequacy of the randomization scheme.

Radiation oncology uses many images of various modalities in the planning, monitoring, and verifying the treatment for patients. An improvement in the technologies of visualization and evaluation of images is likely to have a significant impact on the entire radiation therapy process. Although data presented in this study focus primarily on treatment planning, true 3D image display may enhance our capability in image‐guided procedures for both external beam and brachytherapy. In addition, it may also improve the effectiveness of radiotherapy education for staff and patients. The findings in this paper support continued development of autostereoscopic image display for radiation therapy application.

## Supporting information

Supplementary Material Files
