# Supplementary figures and images for: Application of holographic display in radiotherapy treatment planning II: a multi‐institutional study
Source: J Appl Clin Med Phys. 2009 May 28;10(3):115–24. doi: 10.1120/jacmp.v10i3.2902 (PMC5720557; doi:10.1120/jacmp.v10i3.2902)

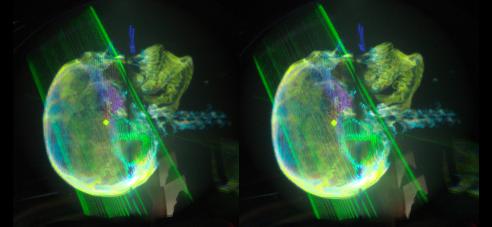

Supplement: Supplementary file 1 — Supplementary Material Files [file ACM2-10-115-s001.JPG]
